# Peri-prosthetic trans-patellar fractures after Total knee Arthroplasty: a case series and review of literature

**DOI:** 10.1186/s42836-020-00050-8

**Published:** 2020-11-04

**Authors:** Gaurav Govil, Lavindra Tomar, Pawan Dhawan

**Affiliations:** 1 Department of orthopaedics, Max Super Speciality Hospital, 108 A, I.P. Extension, Patparganj, Delhi, 110092 India; 2 D-101, Sunshine Helios, Sector 78, Noida, Uttar Pradesh 201305 India; 3 Address: A-702 Vardhman apartment, Mayur Vihar phase1extension, Delhi, 110091 India; 4 Address: House no 37, Sukh Vihar, Delhi, 110051 India

**Keywords:** Peri-prosthetic patella fracture, Patella, Osteosynthesis, Complication, Trans-patellar fracture

## Abstract

**Abstract:**

Peri-prosthetic patella fracture is the second most common peri-prosthetic fracture after total knee arthroplasty. This report presented the treatment results in 6 patients with peri-prosthetic patella fractures. From January 2015 to February 2019, six patients with peri-prosthetic patella fractures were treated. The mean age at surgery was 64 years (range, 48–72 years). Four patients with displaced fractures were treated surgically, and two patients with non-displaced fractures were treated non-surgically. Outcomes were assessed in terms of motion, functional knee score, and Knee Society score. The mean follow-up period lasted 16 months (range: 12–20 months). The average arc of motion was 110° (range: 80°–130°). The mean functional knee score was 77 (range: 70–87). The mean Knee Society score was 84 (range: 75–89). The non-surgical treatment may be a good choice for non-displaced peri-prosthetic patella fractures. For displaced fractures, surgical treatments yielded good functional outcomes.

**Level of evidence:**

IVa

## Introduction

Peri-prosthetic patella fracture (PPPF) represents the second most common peri-prosthetic fracture after total knee arthroplasty (TKA). The reported prevalence rates stood somewhere between 0.2–21% in resurfaced patellae and was about 0.5% in un-resurfaced patellae [1–5]. Most PPPFs occur within 2 years after arthroplasty. Treatments include non-surgical and surgical methods, depending on the features of fractures [2, 4].

Currently, there is no universally-accepted validated classification system for PPPFs. The Ortiguera and Berry classification is most commonly used. It takes into account both stability of patellar implant and the extensor mechanism [6]. Goldberg *et al* [7] also developed a classification on the basis of extensor apparatus continuity and stability of patella resurfacing. However, this classification does not consider variability of fracture configuration and involvement of quadriceps tendon. Trans-patellar fractures with stable implant and intact extensor mechanism can be treated non-surgically [8]. On the other hand, displaced trans-patellar fractures require open reduction and internal fixation [5]. Patellar fractures with unstable implant require a revision arthroplasty.

This report introduced the treatments of PPPFs, with a review of the literature conducted.

## Patients and methods

Informed consent was obtained from each patient before treatments. From January 2015 to February 2019, six patients with PPPFs were treated in our hospital, including two males and four females. The mean age at surgery was 64 years (range, 48–72 years). The trans-patellar fractures occurred, on average, 14 months (range: 2 to 24 months) after TKA. Against the Ortiguera and Berry classification, 3 were of type I and 3 type II. Combined rheumatoid arthritis was found in one patient, and combined osteoarthritis was found in the other five patients. Post-traumatic PPPFs occurred in four knees, and atraumatic PPPFs were found in two knees. Four patients had pain. The PPPFs occurred on the left knee (*n* = 4) and right knee (*n* = 2) (Table 1). The index TKA was performed through the medial parapatellar incision. The prostheses included cemented posterior stabilized press-fit condylar implants (Sigma, DePuy Orthopaedics Inc., IN, USA) (*n* = 4) and implants with a tibial stem extender and a high-flexion rotating platform (Sigma, DePuy Orthopaedics Inc., IN, USA) (*n* = 2). There were two resurfaced patellae and four non-resurfaced patellae. We used a three-peg oval polyethylene patella for resurfacing. All treatments were performed by the same senior surgeon (LT). Two type I PPPFs were treated non-surgically, and the remaining four PPPFs (one type I and three type II) were treated surgically. After surgery, early mobilization with brace or plaster support was used whenever possible. The operated knees were immobilized for 4 weeks, and non-operated knees for 6 weeks. Range of motion exercise was started thereafter. Progressive knee flexion was advised based on the radiological evidence of fracture healing and clinical assessment of quadriceps muscle strength. Three months after treatment, patients were allowed to walk without walking aids.

**Table 1:** Details of 6 patients with peri-prosthetic patella fractures

Case	Age (year)	Sex	Side	Implant	Cause	Association	Surface	TFITT (month)	^a^Type	Treatment
1	68	F	L	Rotating platform PS knee	Fall	OA	Re	2	II	ORIF- CW + SE
2	65	F	L	PFC Sigma	Fall	OA	NRe	23	II	ORIF- SE + bracing
3	72	F	R	PFC Sigma + tibial stem	RRC	OA	NRe	8	I	ORIF – TBW + SE
4	64	F	L	PFC Sigma	RTA	OA	NRe	24	I	Cast
5	48	M	L	PFC Sigma	No trauma	RA	Re	15	I	Cast
6	69	M	R	PFC Sigma	No trauma	OA	NRe	12	II	ORIF – TBW + SE
Mean	64							14		

*RA* Rheumatoid arthritis, *OA* Osteoarthritits, *Re* Resurfaced patella, *NRe* Non-resurfaced patella, *RRC* Rising from a chair, *TFITT* Time from injuries to treatments; ^a^Ortiguera and Berry classification; *ORIF* Open reduction and internal fixation, *CW* Cerclage wiring, *TBW* Tension band wiring, *SE* Suturing with Ethibond

Outcome assessments covered anterior knee pain, extensor lag, arc of motion, and functional ability (Excellent: arc of motion > 110°, extension lag < 5°, no pain; Good: arc of motion = 80° –110°, extension lag = 5–10°, mild pain; Fair and Poor: arc of motion < 80°, extension lag > 10°, severe pain) [5]. The knees were also assessed in terms of Knee Society score.

## Results

**Fig. 1:**
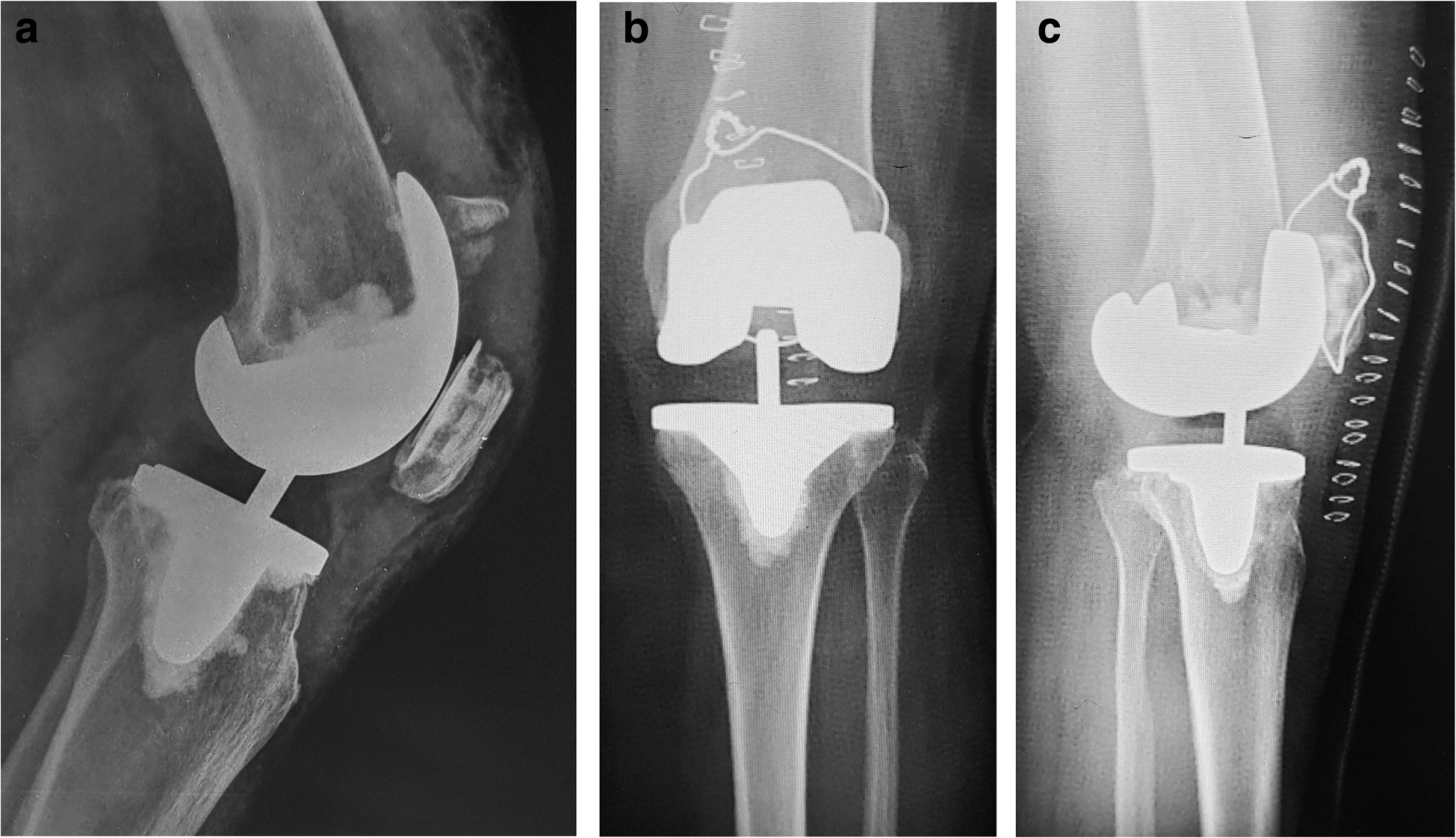
A 68-year-old female patient who suffered a peri-prosthetic patella fracture (PPPF) in her left knee (case 1 in Table 1). **a** Preoperative lateral X-ray shows a PPPF with extensor mechanism rupture and rotating hinge knee system (Depuy) with stable patellar implant. **b** Postoperative anteroposterior view shows the fracture is reduced and fixed with cerclage wiring. **c** Lateral X-ray shows good approximation

**Fig. 2:**
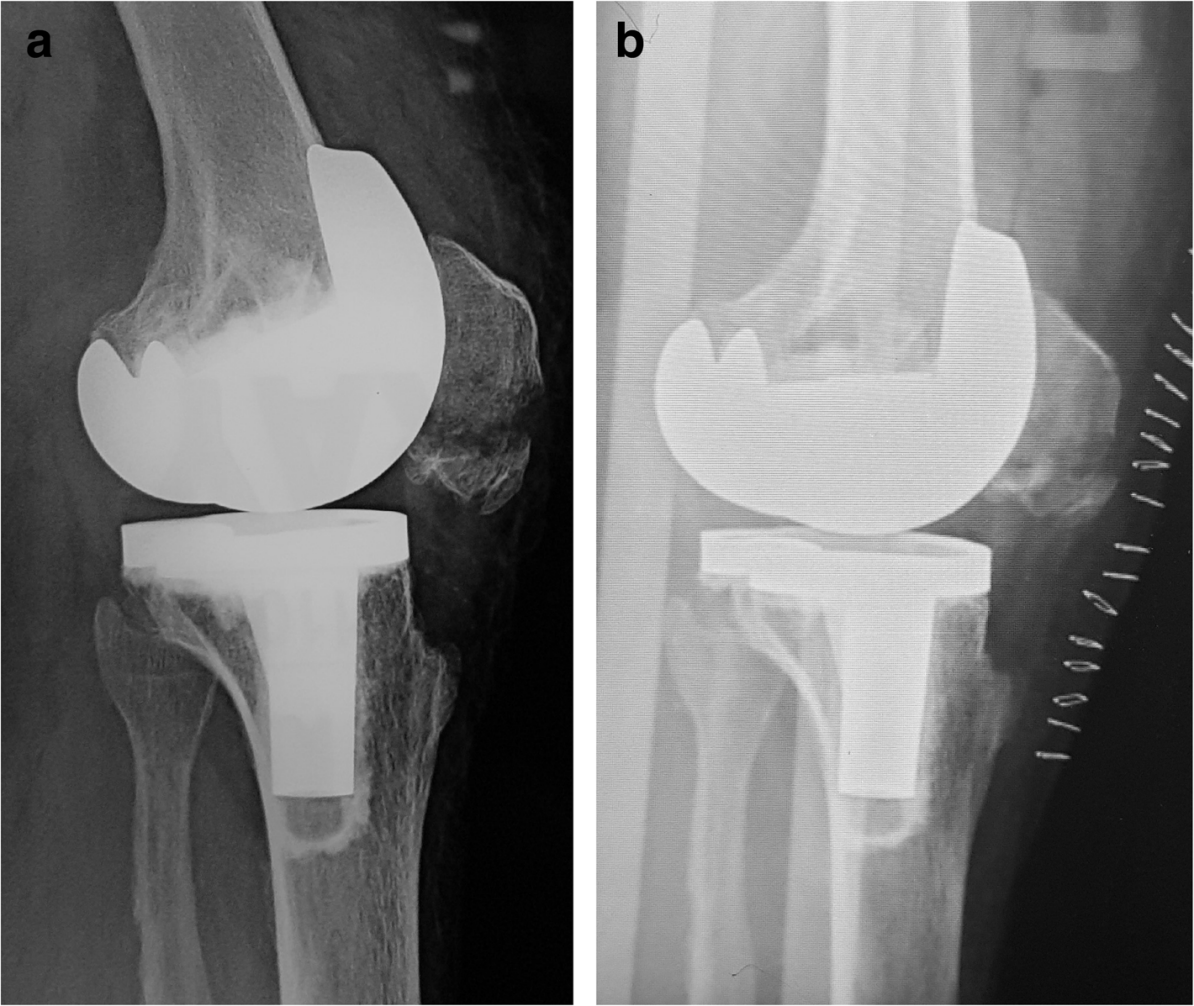
A 65-year-old female patient who had a PPPF in her right knee (case 2 in Table 1). **a** Preoperative lateral X-ray shows the displaced and comminuted PPPF with extensor mechanism rupture. **b** Fixation is achieved with Ethibond polyester suture

**Fig. 3:**
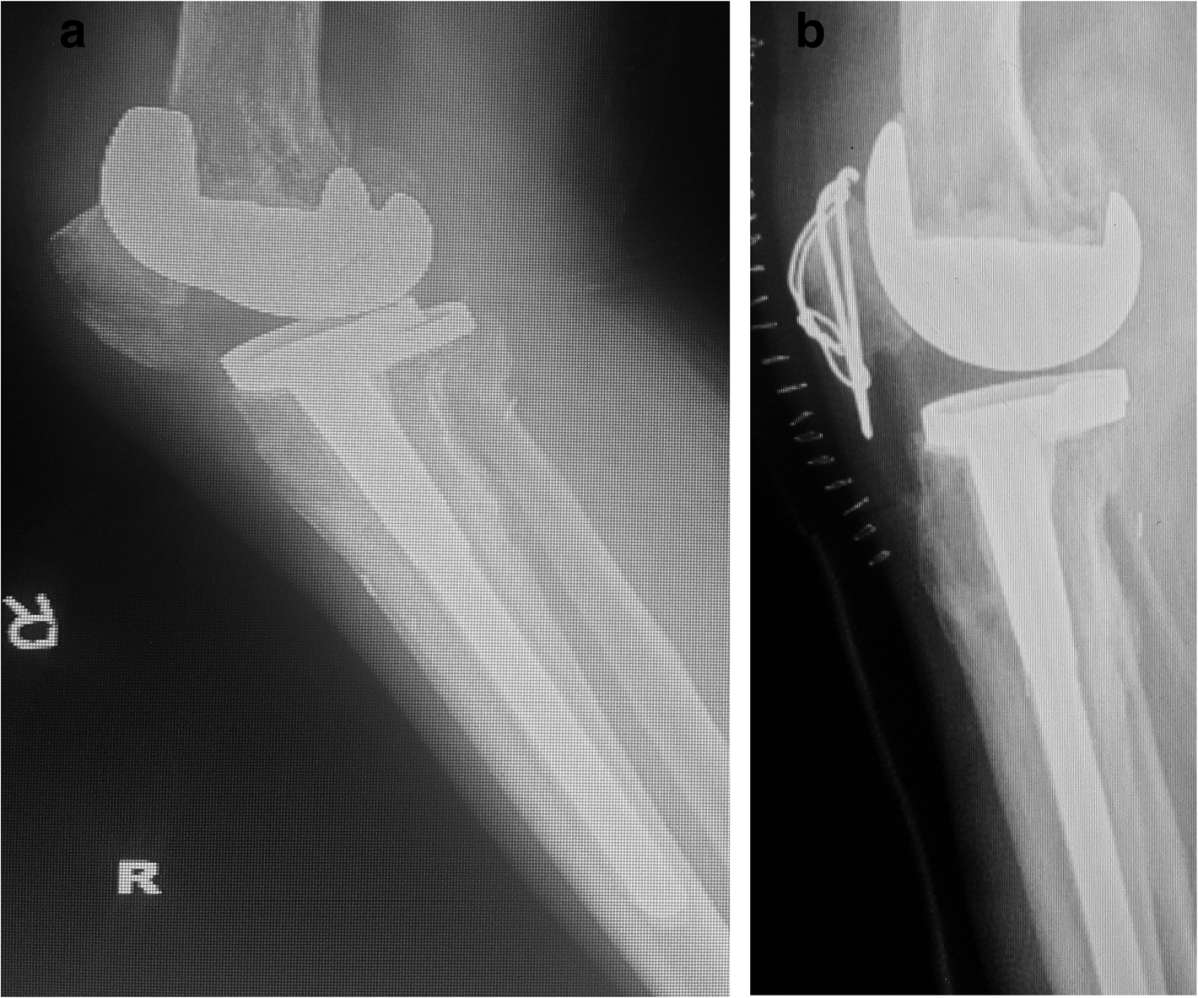
A 72-year-old women who suffered a PPPF (case 3 in Table 1). **a** Preoperative lateral view showing a posterior stabilized system (Depuy) with stem extender. **b** The displaced inferior pole fracture is fixed with tension band wiring

**Fig. 4:**
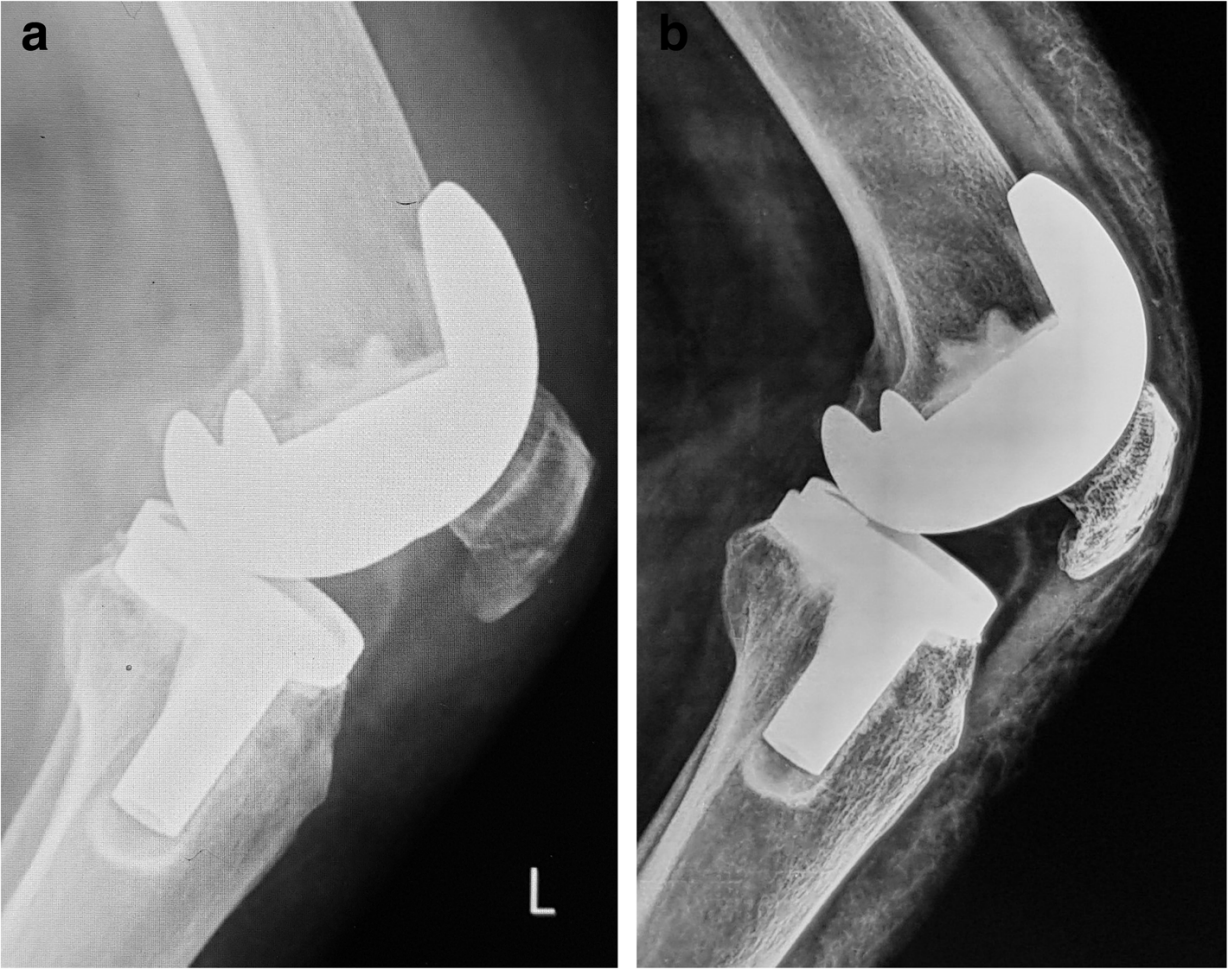
A 64-year-old female who suffered a PPPF in her left knee (case 4 in Table 1). **a** Preoperative lateral X-ray shows a posterior stabilized system (Depuy) and minimally displaced fracture. **b** The fracture is managed conservatively with good union one year later

There was no reoperation. None of the patients developed infection, deep vein thrombosis, or pulmonary embolism. The mean follow-up period lasted 16 months (range: 12–20 months) (Table 1). The average arc of motion was 110° (range: 80°–130°). The functional knee score was 77 (range: 70–87). The mean Knee Society score was 84 (range: 75–89) (Table 2). The findings of pre- and postoperative X-ray examination of implants are presented in Figs. 1, 2, 3, 4.

**Table 2:** Outcomes after one year

Case	Follow-up (month)	AOM (°)	Extensor lag (°)	Outcome	KSS	Functional score
1	18	110	10	Good	84	75
2	15	100	10	Fair	83	70
3	18	80	20	Poor	75	70
4	20	130	0	Excellent	89	87
5	12	120	5	Excellent	89	86
6	15	110	10	Good	86	76
Mean	16	108	9		84	77

*AOM* Arc of motion, *KSS* Knee society score

## Discussion

### Epidemiology

PPPFs usually occur postoperatively and intraoperatively [9]. We conducted a comprehensive systematic review till 2006, and found that the majority of PPPFs was of type III (55%), followed by type I (25%) and type II (20%) [10]. In our series, traumatic events accounted for 2/3 of PPPFs, and two PPPFs were asymptomatic. The female-to-male ratio was reverse probably because increasing TKA was performed in elderly female patients who had a combined osteoporosis.

### Risk factors

The risk factors of PPPFs include advanced age, osteoporosis, over-clamping of the patella during resurfacing, over-reaming of the patella, slippage of the reamer, aggressive patella resection with remaining bone stock less than 10 to 15 mm, thermal injury, bone necrosis due to polymethylmethacrylat cement, and revision of the patellar component particularly in patients with less bone stock [2–4]. Resurfaced patellae are more prone to fracture than their non-resurfaced counterparts [3]. Either under-correction or over-correction should be avoided [11]. A thicker patella may cause the loss of flexion and lateral subluxation, whereas a thinner patella may result in patellar stress fracture and anteroposterior instability of the knee [4, 5, 10]. During lateral release, preserving the lateral vessels, superior lateral genicular artery, and the fat pad can decrease patellar devascularisation [2–4, 10]. Associated medical comorbidities, including rheumatoid arthritis, diabetes mellitus, chronic renal failure, obesity, and hyperthyroidism, may be associated with poor outcomes [1, 4–7].

During the primary TKA, it is important to select a fitting patellar component, correctly position the components, and achieve proper patellar match and tracking. Larger femoral components and mal-positioning of the femoral components in flexed position increase the reaction force of patellofemoral joint, resulting in an elevated risk for PPPFs [9]. Other issues that deserve our attention include: (1) a posterior-stabilized total knee prosthesis had increased contact stress across the femoral component, thereby increasing the patellofemoral contact stresses and the risk of patellar fracture [9]; (2) using a single large central peg component may disrupt the intraosseous vascular supply [4]; (3) revision TKA may be an independent risk factor, which increases possibility of immediate post-operative fractures [1].

### Clinical manifestations

Most PPPFs are asymptomatic, and often have underlying pre-existing factors causing aseptic loosening, infection, arthrofibrosis, and patellofemoral complications [1–5, 9, 10, 12]. Patients with missed injuries may suffer from instability of the knee joint, followed by the failure of TKA [8]. In patients highly suspected of the injuries, extensor mechanism ruptures and fractures should be ruled out.

### Imaging study

X-ray examination usually suffices to identify PPPFs. A skyline view may be helpful sometimes. However, X-rays may not provide definitive evidence of component stability [11]. CT scan shows a better fracture geometry [1]. Technetium TC^99^^m^ medronate bone scan may be useful in the differentiation between old and new fractures [9].

### Treatment selection

Stable and undisplaced trans-patellar fractures are usually treated conservatively. Unstable and displaced trans-patellar fractures may require an internal fixation. If the implant is unstable, a revision surgery is needed [1, 3, 4, 13]. After reviewing 19 studies regarding PPPFs, Chalidis *et* *al* [10] found that 67% of PPPFs were treated non-surgically. They emphasized that surgical revision is required only if there is an injury to the extensor apparatus or patellar implant loosening [1].

We conducted a review of articles published between 2006 and 2020. We found high failure rates (approximately 92%) of surgeries for PPPF. Therefore, simple open reduction and internal fixation were not routinely recommended [2, 10]. Surgical procedures should include restoring continuity of the extensor mechanism by excising small poor-quality osseous fragments, and repairing the remaining extensor tendon [1, 2, 11]. Internal fixation can be achieved using anchor sutures [14], tension wire [15], lag screws with neutralizing plate [16], and locked mesh plates [17]. However, tension-band wiring is desirable for this purpose. Excising the displaced distal pole fragment followed by patellar tendon repair and even patellectomy is indicated if all other treatments fail [12]. Currently, no optimal technique is available for open reduction and internal fixation due to the fact that only limited cases of PPPFs were reported. Usually, superior or inferior pole fractures are treated with tension band technique. If the passive flexion is less than 75° with tension, augmentation of the extensor mechanism using a semi-tendinosus or iliotibial band tendon autograft, allograft, or xenograft is indicated [8, 9].

### Expected outcomes

Good functional outcomes are often achieved in patients without extension lag and with sufficient bone stock [2, 4]. Poor outcomes may be attributed to the coexisting osteoporosis, especially in elderly female patients with combined rheumatoid arthritis. In order to prevent PPPFs, it is important to follow the basic principles of TKA, i.e., achieving the proper extensor mechanism alignment, balancing the soft tissues, and obtaining accurate bone cuts [4, 5, 12, 18]. Type II PPPFs are associated with high rates of complications (50%) and recurrent surgeries (42%) after osteosynthesis. Rehabilitation plays an important role in regaining the pre-fracture level of activity. When removing the patella component, leaving an osseous shell may contribute to the development of anterior knee discomfort and crepitus [9]. Generally, extensor mechanism repair often produces poor results.

### Limitations

Our study has some limitations. The study lacks a control group. The sample size of the series was too small and cannot be used to identify the factors for predicting outcomes. The study cannot serve as a fixation guideline for PPPFs. Future studies should be done in larger cohorts, with control groups set.

## Conclusion

Generally, undisplaced PPPFs should be treated non-operatively whenever possible. For displaced fractures, open reduction and internal fixation and repairing the combined extensor mechanism injury can yield good functional outcomes.

## Data Availability

The datasets used and/or analysed during the current study are available from the corresponding author on request.

## Abbreviations

PPPF: Periprosthetic patella fracture
TKA: Total knee arthroplasty
